# AVENUES OF SOCIAL ISOLATION AND HEALTH AMONG OLDER CHINESE MIGRANTS: A STRUCTURAL EQUATION MODELING APPROACH

**DOI:** 10.1093/geroni/igad104.3199

**Published:** 2023-12-21

**Authors:** Amber DeJohn, Michael Widener, Bochu Liu, Xinlin Ma, Zhilin Liu

**Affiliations:** Florida State University, Tallahassee, Florida, United States; University of Toronto, Toronto, Ontario, Canada; Tongji University, Shanghai, Shanghai, China (People’s Republic); UNC Chapel Hill, Chapel Hill, North Carolina, United States; Tsinghua University, Beijing, Beijing, China (People’s Republic)

## Abstract

Social isolation and loneliness have detrimental impacts on health. During the COVID-19 pandemic, access to third places (e.g., coffee shops, libraries) decreased due to the closure of non-essential destinations for fear of serious illness. With reported rises in anti-Asian hate crimes, which primarily impacted older Asian adults, community mobility might have been additionally affected. Older adults reported adopting ICT during pandemic lockdowns, which may have sufficiently replaced previous activities that would require trips out of the home. Understanding different avenues of social connection and their distinct relationships to health for a vulnerable migrant population is critical to supporting equitable, healthy aging in a post-COVID world. Using a survey of older Chinese migrants in the Greater Toronto Area (GTA) during the extended COVID-19 lockdown, we investigate both community mobility and ICT use to understand how either avenue of socializing is related to the built environment and what the impact of community mobility and ICT use has on loneliness (De Jong Gierveld 6-item scale), mental and physical health (SF-12). Specifically, we use a structural equation model to test a theoretical framework of older adult social isolation. Ultimately, our model demonstrates the importance of community mobility in reducing feelings of loneliness, while ICT use is significantly related to better physical health. Both community mobility and ICT use have significant, although opposite, relationships to transit density. Results indicate that ICT use might have a limited ability to reduce loneliness and support mental health when mobility is limited.

